# P-1424. Patient Experiences at Month 6 after Initiation of Cabotegravir Long-Acting (CAB LA) for PrEP in the First Male Gender Concordant Implementation Science Trial (PILLAR) in the US

**DOI:** 10.1093/ofid/ofae631.1599

**Published:** 2025-01-29

**Authors:** Hadrian Holder, Nanlesta Pilgrim, Roberto Ortiz, William M Valenti, Bo Li, Alison Gaudion, Deanna Merrill, Elizabeth Gibbons, Patrick Daniele, Larissa Stassek, Riya Moodley, Toyin Nwafor, Katherine L Nelson, Kimberley Brown, Maggie Czarnogorski

**Affiliations:** Southwest Community Health Center, Bridgeport, Connecticut; ViiV Healthcare, Durham, North Carolina; Bliss Health, Orlando, Florida; Trillium Health, Rochester, New York; GSK, Collegeville, Pennsylvania; ViiV Healthcare, Durham, North Carolina; ViiV Healthcare, Durham, North Carolina; Evidera, Bethesda, Maryland; Evidera, Bethesda, Maryland; Evidera, Bethesda, Maryland; ViiV Healthcare, Durham, North Carolina; ViiV Healthcare, Durham, North Carolina; ViiV Healthcare, Durham, North Carolina; ViiV Healthcare, Durham, North Carolina; ViiV Healthcare, Durham, North Carolina

## Abstract

**Background:**

Little is known outside of registrational clinical trials about patient experiences on CAB LA, the first long-acting injectable regimen for HIV prevention. We report real-world experiences and outcomes of men at month 6 (M6) after initiating CAB LA in the US.

**Figure:**
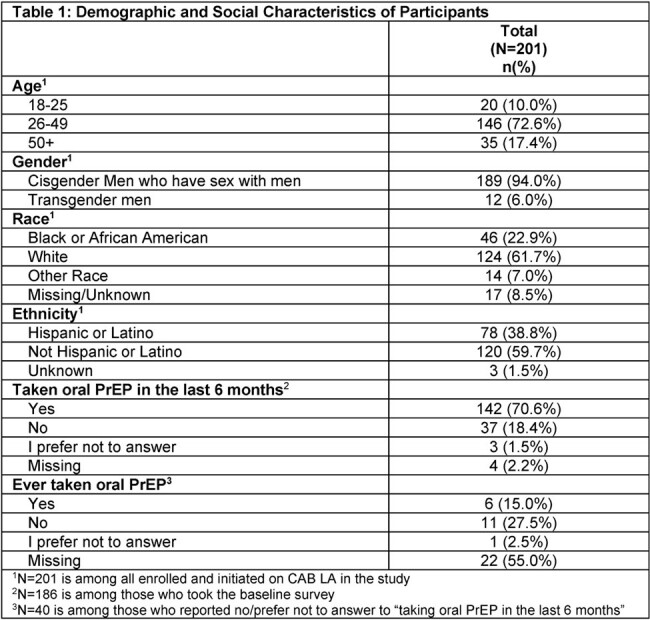

**Methods:**

PILLAR is a Phase 4 gender-concordant implementation science trial assessing integrating CAB LA at 17 clinics for men who have sex with men (MSM) and transgender men (TGM). MSM and TGM completed surveys at baseline (BL) and M6 on experiences and implementation outcomes. A purposeful sample of men completed interviews about their experiences.

**Figure:**
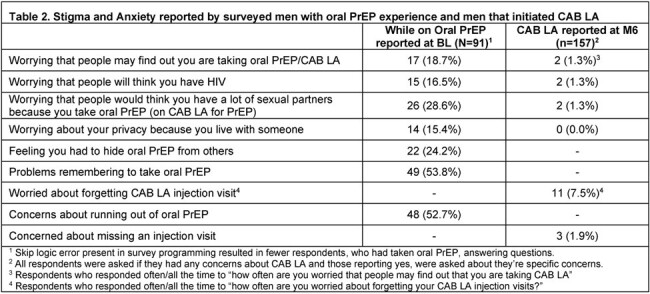

**Results:**

201 men enrolled and initiated CAB LA from May 2022-August 2023, 186 (93%) and 159 (79%) completed BL and M6 surveys, respectively; of these, 45 men completed interviews at M6. Table 1 presents BL demographics. At BL,15-29% of men reported experiencing stigma and anxiety on oral PrEP (Table 2). At M6, 0-1% of CAB LA users reported stigma and anxiety concerns.

At BL, 53-54% oral PrEP users reported concerns about forgetting to take and running out of PrEP. At M6, 2-7% of CAB LA users concerns were forgetting or missing injection visits. Interviewees noted CAB LA reduced stress and fear of missing PrEP, while offering confidence of protection.

High acceptability of CAB LA increased from BL to M6 [mean acceptability: 4.4 (SD:0.69) vs. 4.6 (SD:0.61)] and feasibility remained high [mean feasibility: 4.4 (SD:0.74) vs 4.4 (SD:0.75)]. Interviewees noted CAB LA was convenient for their life. Though 45% reported injection site reactions, majority (86%) returned to daily activities the same day. Few were bothered by injection pain (8%) and reported that pain decreased over subsequent injections.

At M6, 80-92% of survey respondents reported flexible scheduling by clinics as useful to supporting CAB LA use, which was also expressed by most interviewees. Among men who received transportation to visits, had injection reminders and had virtual appointments, over 80% rated them useful.

**Conclusion:**

M6 results show that MSM and TGM report no to little PrEP stigma and anxiety concerns while on CAB LA and that it is feasible and acceptable in their lives. Flexible and virtual appointments and transportation support are helpful while on injectable PrEP.

**Disclosures:**

**Nanlesta Pilgrim, PhD**, GSK: Stocks/Bonds (Public Company)|ViiV Healthcare: Employee **William M. Valenti, MD, FIDSA**, Gilead Sciences: Grant/Research Support|Gilead Sciences: Stocks/Bonds (Public Company)|ViiV Healthcare: Grant/Research Support **Bo Li, PhD**, GSK: Employee **Alison Gaudion, PhD**, GSK: Stocks/Bonds (Public Company)|ViiV Healthcare: Employee **Deanna Merrill, PharmD, MBA, AAHIVP**, GSK: Stocks/Bonds (Public Company)|ViiV Healthcare: Employee **Patrick Daniele, MS**, GSK: Advisor/Consultant|ViiV Healthcare: Advisor/Consultant **Larissa Stassek, MPH**, ViiV Healthcare: Advisor/Consultant|ViiV Healthcare: Travel support **Riya Moodley, FCP**, GSK: Stocks/Bonds (Public Company)|ViiV Healthcare: Employee **Toyin Nwafor, MD**, GSK: Stocks/Bonds (Public Company)|ViiV Healthcare: Employee **Katherine L. Nelson, PhD, MPH**, GSK: Stocks/Bonds (Public Company)|ViiV Healthcare: Employee **Kimberley Brown, PharmD**, GSK: Stocks/Bonds (Public Company)|ViiV Healthcare: Employee **Maggie Czarnogorski, MD MPH**, GSK: Stocks/Bonds (Public Company)|ViiV Healthcare: Employee

